# MiR-184 Mediated the Expression of ZNF865 in Exosome to Promote Procession in the PD Model

**DOI:** 10.1007/s12035-023-03773-2

**Published:** 2023-11-22

**Authors:** Chang Liu, Yang Wang, Jing-wen Li, Xiaoyan Zhu, Hai-song Jiang, Hong Zhao, Li-ming Zhang

**Affiliations:** 1 Department of Neurology, First Affiliated Hospital of Harbin Medical University, Harbin, 150080 Heilongjiang Province China; 2 Department of Neurology, Fourth Affiliated Hospital of Harbin Medical University, Harbin, 150001 Heilongjiang Province China; 3 Laboratory of Basic Medicine, The General Hospital of Western Theater Command, Chengdu, China

**Keywords:** Parkinson’s disease, Exosome, Pathological protein, miR-184

## Abstract

Exosomes are nanoscale small vesicles (EVs) secreted by cells that carry important bio information, including proteins, miRNAs, and more. Exosome contents are readily present in body fluids, including blood, and urine of humans and animals, and thereby act as markers of diseases. In patients with Parkinson’s disease (PD), exosomes may spread alpha-synuclein and miR-184 between the cells contributing to dopaminergic neuronal loss. In this study, we detected the levels of miR-184 in urine-excreted neuronal exosomes between PD patients and age-matched healthy subjects by qRT-PCR analysis. Transmission electron microscopy (TEM) and nanoparticle tracking analysis (NTA) were also used to determine the ultracellular structures of exosomes nanoparticles. MPP + and MPTP were used to construct the cell and animal PD model. Behavioral tests were used to detect motor performance. Furthermore, the cytological experiments were measured to examine the relationship between miR-184 and ZNF865. We found that the levels of miR-184 in urine-derived neuronal exosomes from PD patients were higher, compared to aged-matched normal people. The exosomes from PD patients were larger with greater numbers than those from the age-matched healthy subjects. The difference in miR-184 in urinary exosomes between PD patients and normal people may provide a novel perspective for early diagnosis of PD. However, no difference in CD63 level was observed in Exo-control and Exo-PD groups (exosome from control or PD groups). Moreover, ZNF865 was detected as the targeted gene of miR-184. In addition, miR-184 ASO (miR-184 antisense oligodeoxynucleotide, miR-184 ASO) could rescue the damage of neuronal apoptosis and motor performance in PD mice. Our results showed the miR-184 potential to function as a diagnostic marker of PD.

**Graphical Abstract:**

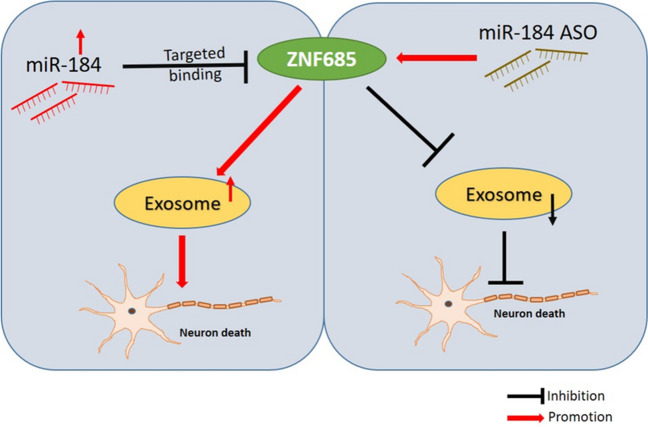

**Supplementary Information:**

The online version contains supplementary material available at 10.1007/s12035-023-03773-2.

## Introduction

Parkinson's disease (PD) is the second most common neurodegenerative disorder after Alzheimer’s disease (AD), which is characterized by tremors and motor retardation and affects about 1.5% of the population ages 65 years and older [1–3]. PD predominately affects dopamine-producing (“dopaminergic”) neurons in the substantia nigra [4–6]. The progression of PD symptoms is usually different from one patient to another due to the diversity of the disease, and the symptoms generally develop slowly over the years. The cause of PD remains largely unknown. Although there is no cure, treatment options vary and include medications and surgery [3, 7]. While Parkinson’s itself is not fatal, disease complications can be serious.

Extracellular vesicles (EVs) are vesicle-like bodies with a bilayer membrane structure that shed from or are secreted by cells, and these vesicles are served as “carriers” for cell-to-cell communication, responsible for the transfer of substances between different cells [8, 9]. EVs are also produced in other types of human tissues (as well as other animals and plants) and represent a relatively new field of research in neurodegenerative disease [10, 11]. EVs are thought to spread in the brain to cause neurodegenerative pathologies and manifestation of diseases, such as PD [12–14]. EVs carry different cargoes, exhibit dynamic changes in number and content in response to physiologic and environmental conditions, and may function as shuttles for the delivery of cargo between cells [15, 16]. Lipids, proteins, second messengers, mRNA, miRNA, and cell organelle fractions embedding in EVs can transfer to target cells by ligand/receptor interaction, fusion, and/or internalization [17]. There is increasing evidence that EVs can contribute to the onset and progression of neurodegenerative and neuroinflammatory diseases and play a key role in various diseases, including PD [18, 19].

Exosomes are important intercellular messengers and significant contributors to both health and disease [20, 21]. Exosomes are important vehicles in CNS [22]. With their nanometric size EVs can travel across the endothelial cells of the blood–brain barrier (BBB) by receptor-mediated endocytosis and release their contents into the biological fluids [23, 24]. EVs can mediate various signaling functions and can be implicated in brain disorders, such as a-syn [1], catalase [25], GDNF [26], miRNA-155 [27], and miRNA-29a [28].

Diagnosis of CNS diseases needs to collect T urine or blood biomarkers would be more efficient for clinical diagnosis and screening, due to fewer limitations of volume. Exosomes can freely cross the BBB and enter the bloodstream, and then enter the urine through the kidneys [29]. Thus, urinary exosomes can reflect the changes in the brain’s environment, at least to a certain degree. Exosomal contents, including nucleic acids, protein, mRNA, and miRNA, could change under disease conditions, and the membranes of the EVs protect their contents from degradation [30]. These characteristics of exosomes favor them as a valuable diagnostic tool. However, it is still a challenge to study exosomes and use them as a diagnostic tool for PD due to their comparatively small size.

MicroRNA (miRNA) is a small non-coding RNA that regulates gene expression after transcription and serves as a potential marker for the diagnosis of diseases [22]. MiR-184 targeting inhibits the invasion, migration, and metastasis of cancer cells in nasopharyngeal carcinoma and breast cancer [31, 32]. Moreover, it has been found that miR-184 is widely expressed in the central nervous system and plays an important role in neuron regulation [33]. However, the role of miR-184 in PD was rarely reported.

To explore the new tool for PD diagnosis, in this study, we aim to explore the role of miR-184 in urine exosomes of PD patients. We used qRT-PCR to detect the levels of miR-184 in urine-derived neuronal exosomes as well as applied transmission electron microscopy (TEM) and nanoparticle tracking analysis (NTA) to characterize the sizes and numbers of the urinary exosomes in PD (and matched controls of healthy subjects with similar ages).

## Materials and Methods

### Patients

All samples were collected from the First Affiliated Hospital of Harbin Medical University. The number of 20 patients were diagnosed with PD on the basis of standard clinical and biochemical parameters according to the latest diagnostic criteria for Parkinson’s disease. Twenty normal controls were healthy donors matched with PD patients in terms of age, region, education, and gender. Exclusion criteria are as follows: exclude frontotemporal dementia, trauma, infection, epilepsy, vascular and other types of dementia based on medical history, magnetic resonance, and CT examination. The Parkinson’s disease diagnosis flowchart was shown in supplement Fig. 1. All subjects were free from another systemic diseases such as diabetes, hypertension, and kidney disease. Informed consent was sought from patients and matched healthy donors prior to the collection of their urine. The clinical data of the patients with Parkinson’s disease and the healthy test groups are shown in Table 1. Consenting patients who met the inclusion criteria and healthy donor donated their urine for study at the outpatient clinic of First Affiliated Hospital of Harbin Medical University. The clinical manifestations of PD in the patients, all of whom were in the moderate to severe stages of disease progression, were characterized by varying degrees of tremor, slowed movement, rigid muscles, impaired posture and balance, loss of automatic movements, and speech and writing changes. A total of 20 ml urine was collected in centrifuge tubes from all the patients and matched healthy donors and stored at 4 °C for the following exosome isolation.

**Fig. 1:**
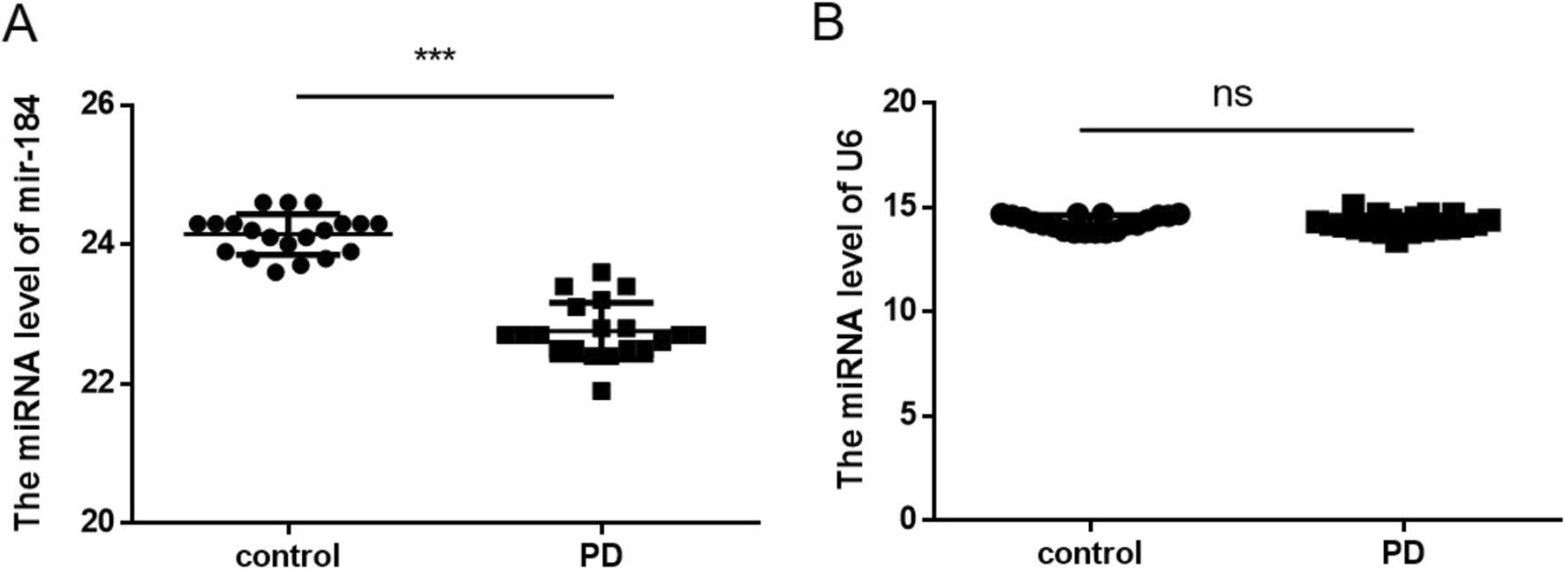
The miRNA levels of miR-184 (**A**) and U6 (**B**) in the urine exosomes were detected by RT-qPCR. Control, urine sample from normal healthy people; PD, urine sample from PD patients. *n* = 20/group. ***p < 0.001

**Table 1:** The clinical data of the patients with Parkinson’s disease and the healthy test groups

Clinical characteristics	PD (*n* = 20)	HC (*n* = 20)	*p*
Gender (man, %)	10 (50%)	10 (50%)	1①
Age (years)	62.55 ± 5.54	61.75 ± 4.75	②
BMI (kg/m^2^)	23.345 ± 2.35	23.415 ± 2.57	-
Duration of symptoms	4.12 ± 2.12	-	-
Age of onset	58.33 ± 2.07		
MoCA score	21.54 ± 4.03	22.01 ± 3.95	②
Hoehn-Yahr stage	1.765 ± 0.70		

PD, Parkinson’s disease; HC, healthy control; ①, chi-squared test; ②, Student *t* test

### PD Animal Model

A total of 18 C57BL/6 J mice (8-week old) were purchased from Chengdu Dossy experimental animal company. Mice were fed in the animal center of Harbin Medical University under specific pathogen-free (SPF). The room constant temperature was 22 ± 0.5 °C with a 12-h light/dark cycle. Mice have free access to water and food. All mice were evenly divided into 3 groups with 6 rats in each group: control, 1-methyl-4-phenyl-1,2,3,6-tetrahydropyridine (MPTP) and MPTP + miR-184 ASO (miR-184 antisense oligodeoxynucleotide, miR-184 ASO). To establish the mouse model of PD, MPTP was injected intraperitoneally with a dose of 30 mg/kg/d for 5 days. PD mice were injected with 2 µl of miR-184 ASO at 0.33 nmol/ml in artificial cerebrospinal fluid (CSF) (Harvard Apparatus, USA) via the intracerebroventricular (i.c.v.) injections. Control mice received the vector in aCSF. All animal experiments were approved by the Institutional Animal Care and Use Committee of Harbin Medical University and followed the recommendations.

### Cell Culture and Treatment

SH-SY5Y cells were cultured in Eagle’s Minimum Essential Medium (EMEM) (30–2003,

ATCC, USA) containing 10% fetal bovine serum (FBS) and 100 units/mL penicillin and streptomycin. The culture medium was changed every 2 days and subcultured when the cell density reached 80%. The PD cell model was established by 1-methyl-4-phenylpyridine (MPP +). SH-SY5Y cells were treated with MPP + (1 mM) or PBS for 12 h, followed by treatment with miR-184 ASO or vector-mixed liquid (5 µg pri-miR-184 ASO pLVX plasmid/vector and 15 µL liposome 2000 were diluted in 750 µL Opti-MEM medium and mixed, and then placed at room temperature for 10 min). The miR-184 ASO were synthesized by Thermo Fisher Scientific. After 48 h, cells were collected for cultured in non-FBS for another 48 h and then the exosome (miR-184-ASO EXO) was extracted.

### TUNEL Staining

The apoptosis in SH-SY5Y cells was analyzed by TUNEL staining as described previously. Cell slices were stained with a commercial TUNEL assay kit (Abcam; ab206386). Nuclei are stained with DAPI solution (Abcam; ab228549). There are three different fields for every section and four sections per group to analyze.

### Immunohistochemistry (IHC)

Tyrosine hydroxylase (TH)-positive cells were detected by IHC. Brain tissue slices (5 µm) were incubated with 3% H_2_O_2_ to remove endogenous peroxidase activity, followed by blocking with blocking buffer. Slices were incubated with anti-TH antibodies (ab6211, 1; 1000, Abcam) overnight at 4 °C and then incubated with secondary antibodies rabbit IgG (ab205718). Next, slices were incubated DAB (D3939, Sigma-Aldrich, USA). The image was captured using a light microscope (Leica DM4000) at × 100 magnification. Finally, the results were analyzed by counting the number of positive cells on a bright microscope.

#### Urinary Exosome Isolation

The urinary exosome was isolated as described previously [34]. In brief, 20 ml urine was centrifuged at 3000xg for 10 min to remove cells and cell debris. The supernatant was mixed with 12.5 ml ExoQuick Exosome Precipitation (ExoQuick-TC) in a sterile tube and incubated at 4 °C for 30 min. Following centrifugation at 10000xg for 30 s at 4 °C, the exosomes appeared at the bottom of the tube. The supernatant was aspirated without disturbing the precipitated exosome pellet, and the exosome was re-suspended in the preferred buffer for the following experiments.

#### Quantitative Real-Time Polymerase Chain Reaction

Total RNA was isolated from urinary exosome samples using the RNeasy Micro Kit (Qiagen) and was reverse transcribed with ASO-dT primers and Quantiscript reverse transcriptase (Invitrogen) in a final volume of 20 µl for 50 min at 42 °C. MiR-184 primers used for PCR amplification were 5′- (forward) and 5′- (reverse). For quantitative analysis of miRNA abundance, the resulting complementary DNAs were PCR amplified in a Rotor-Gene Q (Qiagen) with Platinum SYBR Green qPCR SuperMix-UDG (Invitrogen). Quantification of miR-184 expression was obtained using Rotor-Gene Q Series Software. Relative units estimated from the quantification represent the ratio between specific microRNA molecules and U6 in each sample.

miR-184 primer: F: 5′-CAGAGGGGCTTTGAATTTGA-3′; R: 5′-CCCATCACGCAAGTGCAG-3′

U6 primer: F: 5′-CTCGCTTCGGCAGCACA-3′; R: 5′-AACGCTTCACGAATTTGCGT-3′

#### Western Blotting (WB)

The protein levels were detected as the previously performed [35]. The exosome samples (exosome from control or PD groups, Exo-control or Exo-PD) were lysed in lysis buffer containing 50 mM Tris–HCl, 150 mM NaCl, 1 mM EDTA, 0.5% NP-40, and 1 mM PMSF with protease inhibitor mixture, pH 8.0. The supplement was collected after being centrifugated at 14,000 g for 10 min at 4 °C. The loading buffer was added to the protein samples and boiled for 5 min at 98 °C. The protein samples were resolved by 12% SDS-PAGE and then transferred to PVDF membranes for immunoblotting. PVDF membranes were blocked in blocking buffer (1 X TBS containing 5% skim milk) at room temperature for 1 h, followed by diluting with primary antibodies and HRP-conjugated secondary antibodies. The immunoblotting bands were visualized with an ECL kit (Sangon). Images were captured using a chemiluminescence imaging analysis system (Tanon, 5200) and analyzed with the ImageJ.

Antibodies: Anti-ZNF865 (1:1000, PA5-49280, Thermo), Anti-Caspase 3 (1:1000, 700182, Thermo), Anti-CD63 (1:1000,10628D, Thermo), Anti-GAPDH (1:1000, MA1-16757, Thermo), HRP-conjugated goat anti-mouse or anti-rabbit IgG secondary antibodies (1:5000, Proteintech).

### Loading miR-184 ASO into Exosomes

The miR-184 ASO was loaded to exosome as previously reported [36]. MiR-184 ASO was synthesized by Thermo Fisher Scientific. MiR-184 ASO was diluted to 0.1 mg/ml in PBS with dextran. The isolated exosome was mixed with miR-184 ASO solution and then introduced into mice at an 8-µl/min flow rate. All collected samples were filtered by an action filter and then the excess dextran was removed with PBS.

#### Transmission Electron Microscopy

Transmission electron microscopy was performed as previously reported [37]. The urinary exosome samples from 10 PD patients and 10 matched healthy controls were investigated by transmission electron microscopy. The exosome pellet was re-suspended in 30 µl PBS and 3 µl of the solution was placed onto a copper net grid with carbon film for the electron microscope. The excess fluid was wicked off after 1 min, and a drop of phosphotungstic acid dye solution was added. After another min, excess fluid was wicked off, and the sample was allowed to air dry. The completely dry copper mesh was photographed using a Philips-FEI Tecnai TEM (FEI T12).

#### Nanoparticle Tracking Analysis

The urinary exosome samples from 10 PD patients and 10 matched healthy controls were investigated by nanoparticle tracking analysis (NTA). The samples from the patients and healthy controls have no significant differences in age, gender, and education levels. The exosome pellet derived from urine was re-suspended in 300 µl PBS. The solution was diluted 50 times before testing using the NanoSight LM10 instrument (NanoSight Ltd., Amesbury, UK), which sends a finely focused laser beam through a glass prism to illuminate the sample (a liquid containing a dilute suspension of particles). The sample was run 3 times, and two videos were captured for 1 min in each run. All analysis settings were kept constant within each experiment. Size distribution profiles obtained from NTA were averaged for each sample across the video replicates and then averaged across samples to determine representative size distribution profiles.

The unique nanoparticle tracking analysis (NTA) technique utilizes the properties of light scattering and the Brownian notion to obtain the particle size distribution and number of particles in a liquid suspension. The laser beam passes through the sample chamber and travels through the particles in the suspension along the path of the beam’s scattered light, making it easy to visualize the particles through a × 20 magnification microscope with a camera. The camera operates at 30 frames per second and captures video files of moving particles under Brownian motion. The software tracks multiple particles individually and uses the Einstein equation to calculate the hydrodynamic diameter of the particles.

### Luciferase Reporter Assay

Database tools EvmiRNA (http://bioinfo.life.hust.edu.cn/EVmiRNA/#!/) and TargetScan (http://targetscan.org/mamm_31/) were used to predict the binding sites between ZNF and the miR-184.

The sequence was cloned into a luciferase expression vector (GenePharma, Shanghai, China) containing firefly luciferase (F-luc). Then, SH-SY5Y cells transfected with ZNF865 promoter region reporter vectors and were further co-transfected with miR-184 in cells. The Dual Luciferase Reporter kit (Promega) was used to detect luciferase activity.

### Behavioral Tests

#### Rotarod Test

As previously described, the rotarod test was used to evaluate the motor performance of mice [38]. The mice were used to stay on the spindle for the test. In 1 min, the speed of the rod was accelerated from 0 to 40 rpm and then maintained a constant speed. The latency to fall (time) was recorded when the mice fell off. Carry out five experiments in a row.

#### Pole Test

Mice were challenged in pole tests to detect motor performance [37]. Mice were adapted to the environment and trained for 2 days before the test. Mice were placed facing up at 5 cm from the top of a pole (diameter 1 cm and length 50 cm). The time in the pole was recorded. Carry out five experiments on each mouse.

### Statistical Processing

Statistical analysis was performed using SPSS Statistics 21.0 software. Two independent sample *t* tests were run for the continuous variables (i.e., the number of exosomes and the level of protein) with normal distribution. The ANOVA was used for analysis among more than three groups. The data obtained were represented by X ± S. *p* < 0.05 was considered statistically significant.

## Results

### MiR-184 in Urine Exosomes from PD Patients Was Higher than That from Age-Matched Healthy Donors

Exosomes carry different cargoes and exhibit dynamic changes in number and content in response to physiologic and environmental conditions and may function as shuttles for the delivery of lipids, proteins, second messengers, mRNA, miRNA, and cell organelle fractions, which can transfer to target cells by ligand/receptor interaction, fusion, and/or internalization [39]. We examined miR-184 abundance in urine-derived neuronal exosomes from PD patients and age-matched healthy donors by quantitative real-time polymerase chain reaction (qRT-PCR). The results showed that the levels of miR-184 were significantly elevated in the urine exosomes of PD patients compared to the levels in the urine exosomes of healthy donors (Fig. 1). These data suggested that the level of miR-184 may indicate the PD disease process and may be used as a biomarker since urine is more easily accessible and more efficient for clinic screening and diagnosis than cerebrospinal fluid.

### Under TEM, Exosomes in Patients with PD Were More Concentrated than in Normal People

**Fig. 2:**
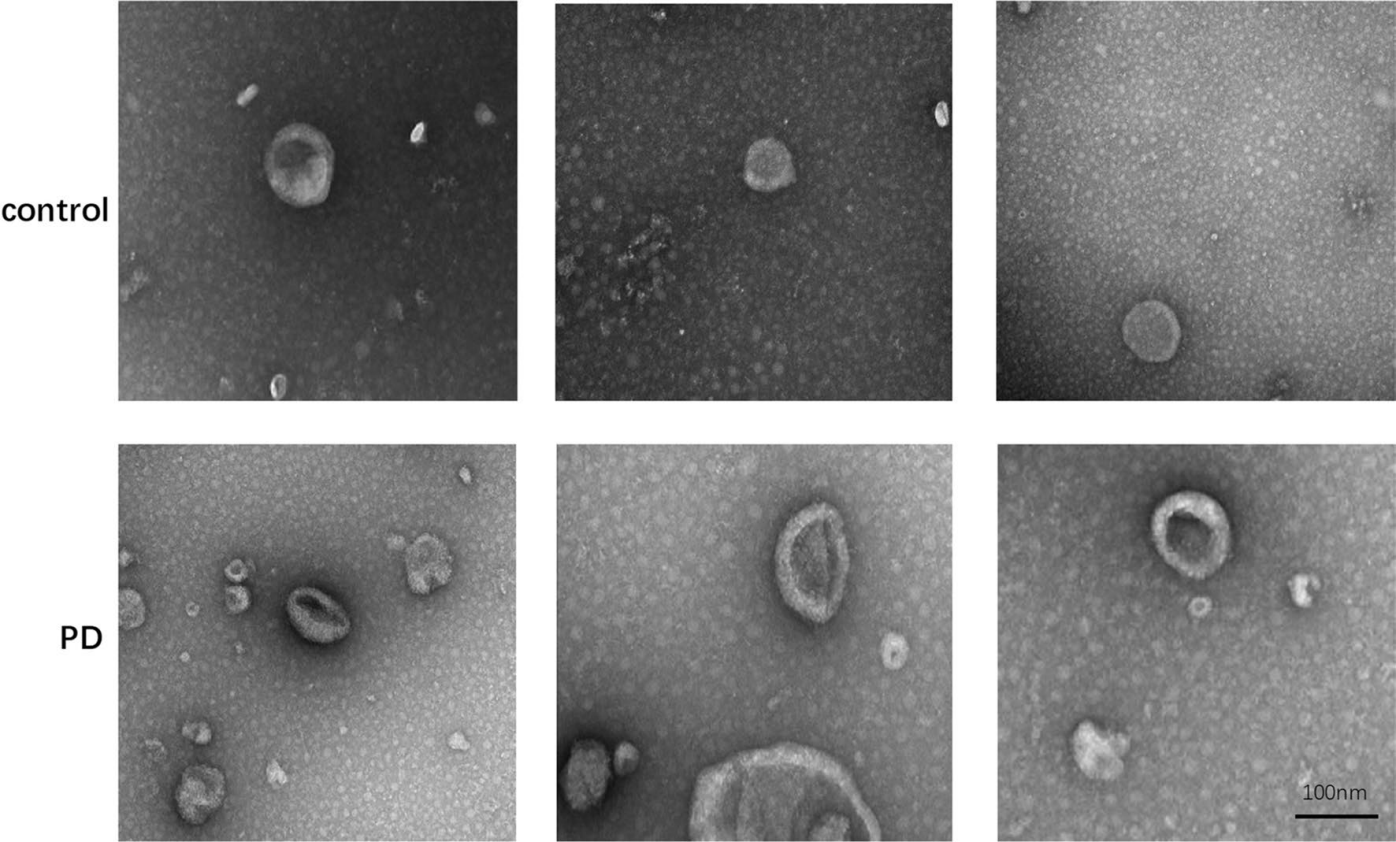
Exosome morphology in control (normal healthy people) and PD with TEM analysis

Ultrastructural analysis by transmission electron microscopy of preparatively isolated urine-derived neuronal exosomes showed that ring-shaped exosomes were detected in both PD patients and the healthy donor group (Fig. 2). The exosomes from PD patients were relatively concentrated compared to those from the matched healthy donors. Previous studies have shown that exosomes may shrink or change size during preparation for TEM [39], however, we did not detect any clear morphological change, and electron microscopy is still useful for investigating the difference between the exosomes from the two groups.

### The Size of the Exosomes from PD Patients Was Larger than the Age-Matched Normal People

We further detect the size of exosome in urine of PD and health donors; the results showed that the exosomes were most abundant in the size range of 30–150 nm, which is consistent with the previous research [40, 41]. Then, the concentration of urine-derived exosome with diameters ranging from 30 to 150 nm was detected; we found that there was 3.04e + 009 ± 3.49e + 007 particles/ml of exosome in PD patients, which was higher than that in healthy donors (the concentration was 2.30e + 009 ± 2.90e + 008 particles/ml); however, the statistically significant difference was not observed (*p* > 0.05).

**Fig. 3:**
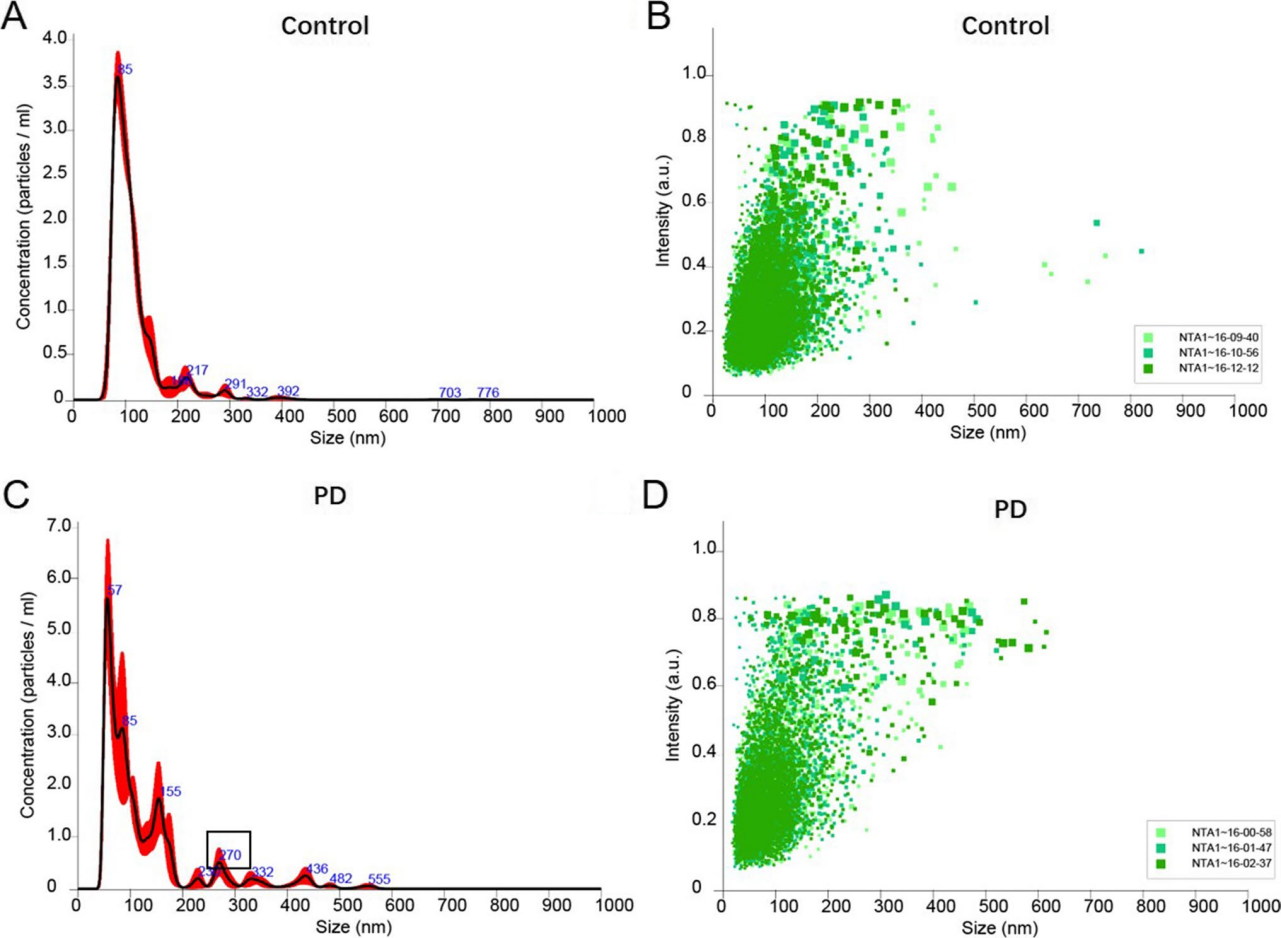
NTA analysis of exosomes of PD patients and healthy controls. **A**, **B** The representative concentration and intensity of exosomes in control groups, respectively. **C**, **D** The representative concentration and intensity of exosomes in PD patients groups, respectively. Control, normal healthy people; PD, PD patients. *n* = 3/group

**Fig. 4:**
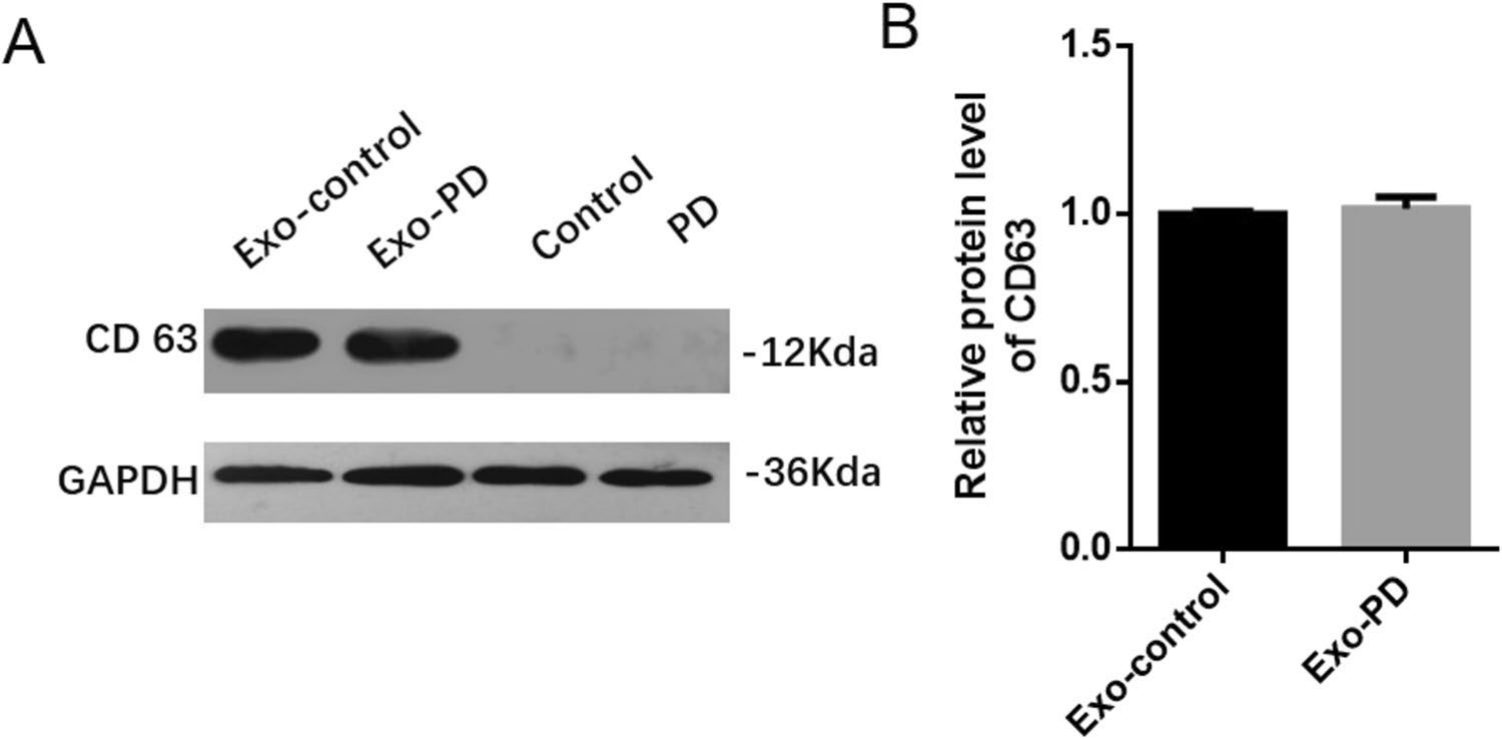
The level of CD63 in exosomes. **A** The protein level of CD63 in exosomes in control and PD groups. **B** The statistics of **A**. Exo-control, exosome sample from cell without MPP^+^ treatment; Exo-PD, exosome sample from cell with MPP.^+^ treatment. *n* = 3

We used the same NTA strategy to examine the distribution of the urine-derived exosomes from PD patients and healthy donors. In these samples, the mean size of exosomes from PD patients was 185.1 ± 1.6 nm and the D10 (the diameter of particle size distribution reached 10%), D50 (the diameter of particle size distribution reached 50%), and D90 (the diameter of particle size distribution reached 90%) were 115.8 ± 2.5 nm, 162.8 ± 0.8 nm, and 281.3 ± 7.9, respectively. Compared to the PD patients, the mean size of exosomes from healthy donors was 157.9 ± 6.1 nm and the D10, D50, and D90 were 97.2 ± 4.4 nm, 142.7 ± 4.9 nm, and 231.7 ± 14.9 nm. We can detect a second peak at a diameter of 272 nm in the exosomes of PD patients (Fig. 3C). However, we did not detect any peaks after the main peak in the exosomes from the healthy donors. These results showed that the size of exosomes from PD patients was larger than that of the age-matched healthy donors (Fig. 3A). The size and concentration difference of the urine-derived exosomes did not correlate with any feature of the disease including sex, age, education level, and region. However, the protein level of CD63, which was an exosome marker, has no difference in PD-derived exosomes compared to control exosomes (Fig. 4). This data indicated that we use the same amount of exosome to detect miR-184.

### Effects of miR-184 ASO on the Expression of ZNF865 and Caspase 3 Protein in the PD Model

**Fig. 5:**
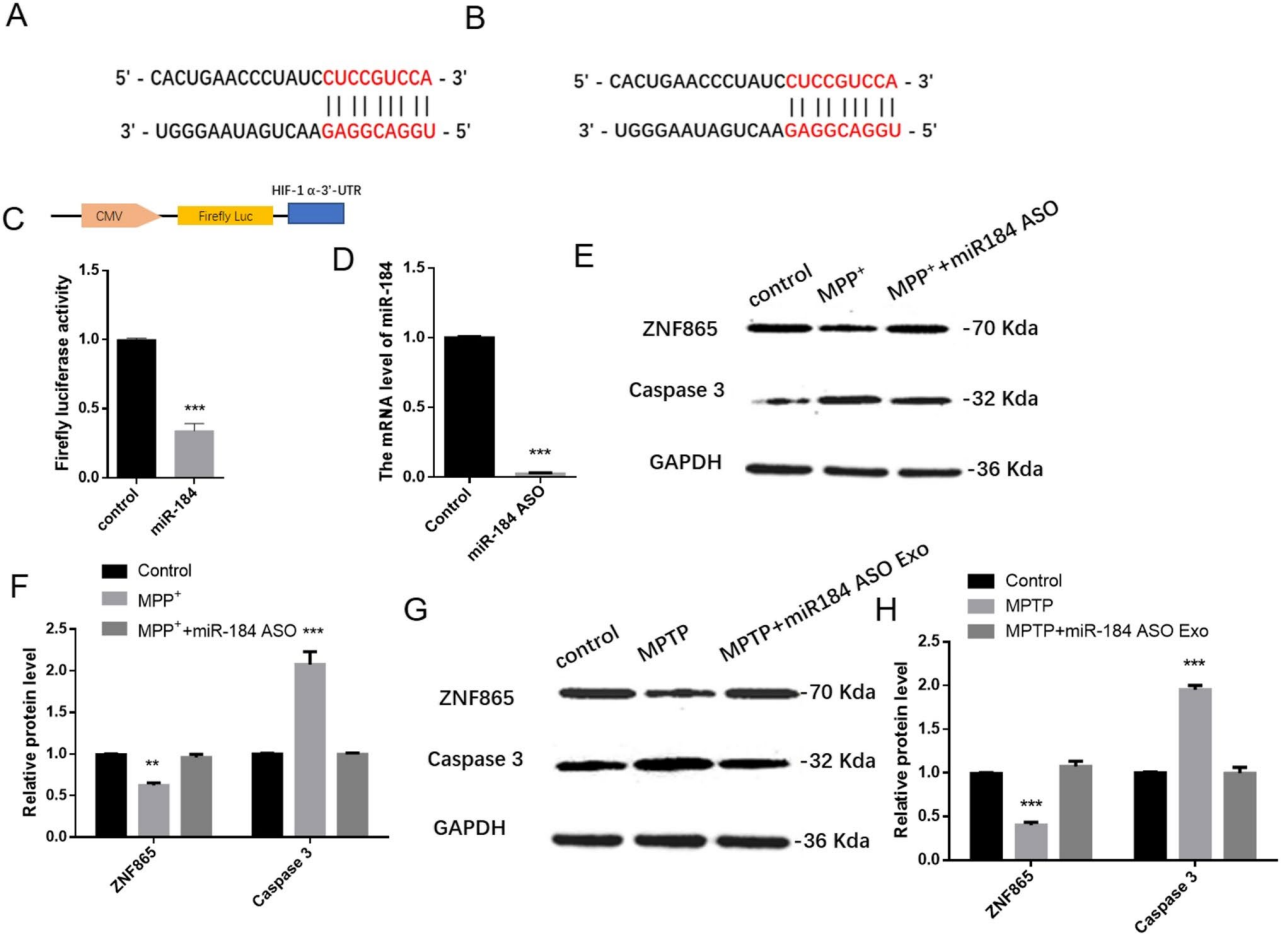
Effects of miR-184 ASO on the expression of ZNF865 and caspase 3 protein in cell and mice model of PD. **A** miR-184 targeted site in ZNF865 mRNA in humans. **B** miR-184-targeted site in ZNF865 mRNA in the mouse. **C** The firefly luciferase activity analysis in MPP + -induced SH-SY5Y cells. **D** The miR-184 level in MPP + -induced SH-SY5Y cells. **E** Western blotting results of cells to verify the expression of ZNF865 and caspase 3 proteins. **F** Quantification of ZNF865 and caspase 3 in **E**. **G** Western blotting verified the expression of ZNF865 and caspase 3 proteins in animal experiments. **H** Quantification of ZNF865 and caspase 3 in **G**. All *n* = 3. **p < 0.01 and ***p < 0.001

To determine the potential role of miR-184 in PD, we used bioinformatics analysis to predict possible targets. The data shows that miR-184 targeted ZNF865 (Fig. 5A and B). ZNF865 was involved in transcriptional regulation and affected in prognosis, immunity, and treatment of esophageal cancer [40]. The luciferase assay also determined the interaction between miR-184 and ZNF 865(Fig. 5C). The miR-184 level was highly expression in miR-184 ASO (Fig. 5D).After MPP + treatment, the expression of caspase 3 increased in cells (Fig. 5E and F), indicating that MPP + treatment induced the activation of the apoptosis pathway and successfully mimicked PD cells. Compared with control group cells, the ZNF865 protein level decreased in the MPP + group, which is consistent with the results of exosomes of PD mice (Fig. 5G and H). MiR-184 ASO lowered caspase 3 levels and increased ZNF865 levels in MPP + treated cells, suggesting that ZNF865 protein can alleviate the damage caused by PD and therefore have nerve-protective effects. Similar results were also confirmed in MPTP-induced PD animal model (Fig. 5G and H).

### The Injection of miR-184 ASO Exosomes Alleviated Experimental Apoptosis in MPP + -Induced Cell Models

**Fig. 6:**
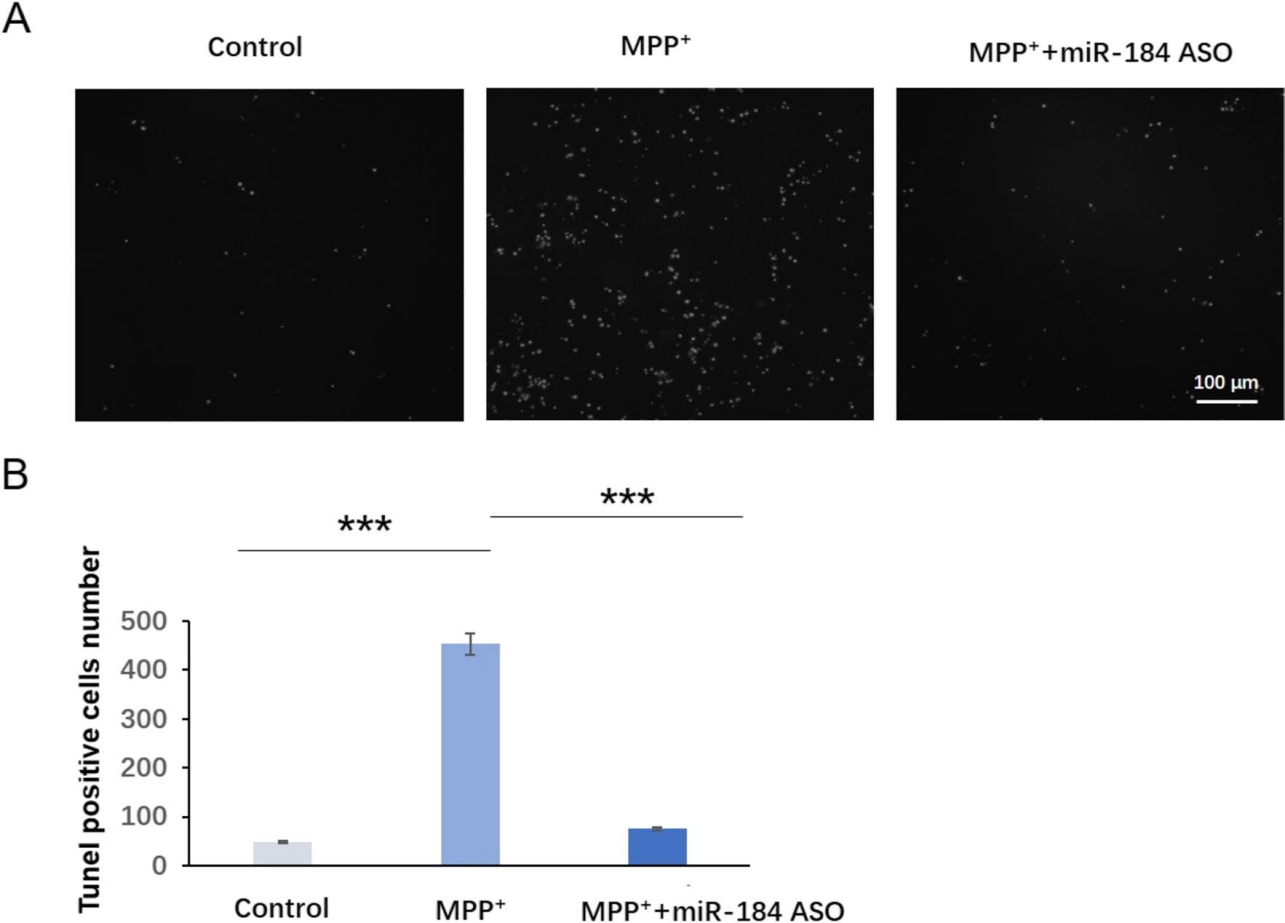
The apoptosis level in MPP.^+^-induced cell model. **A** The comparison of experimental neuronal apoptosis. White, positive TUNEL signal representing apoptotic cells. **B** Results of TUNEL positive cell number analysis for each group. *n* = 3. ***p < 0.001

The TUNEL assay is used to detect apoptosis of each group of nerve cells (Fig. 6 A and B). The number of apoptotic cells in the MPP + group increased significantly compared to the control group. MiR-184 ASO significantly inhibited MPP + -induced apoptosis, confirming the protective effect of miR-184 ASO on nerve cells against MPP + damage.

### Injection of miR-184 ASO Exosomes Can Alleviate the Damage of Dopamine Neurons in the Brain Mice of the Control Group

**Fig. 7:**
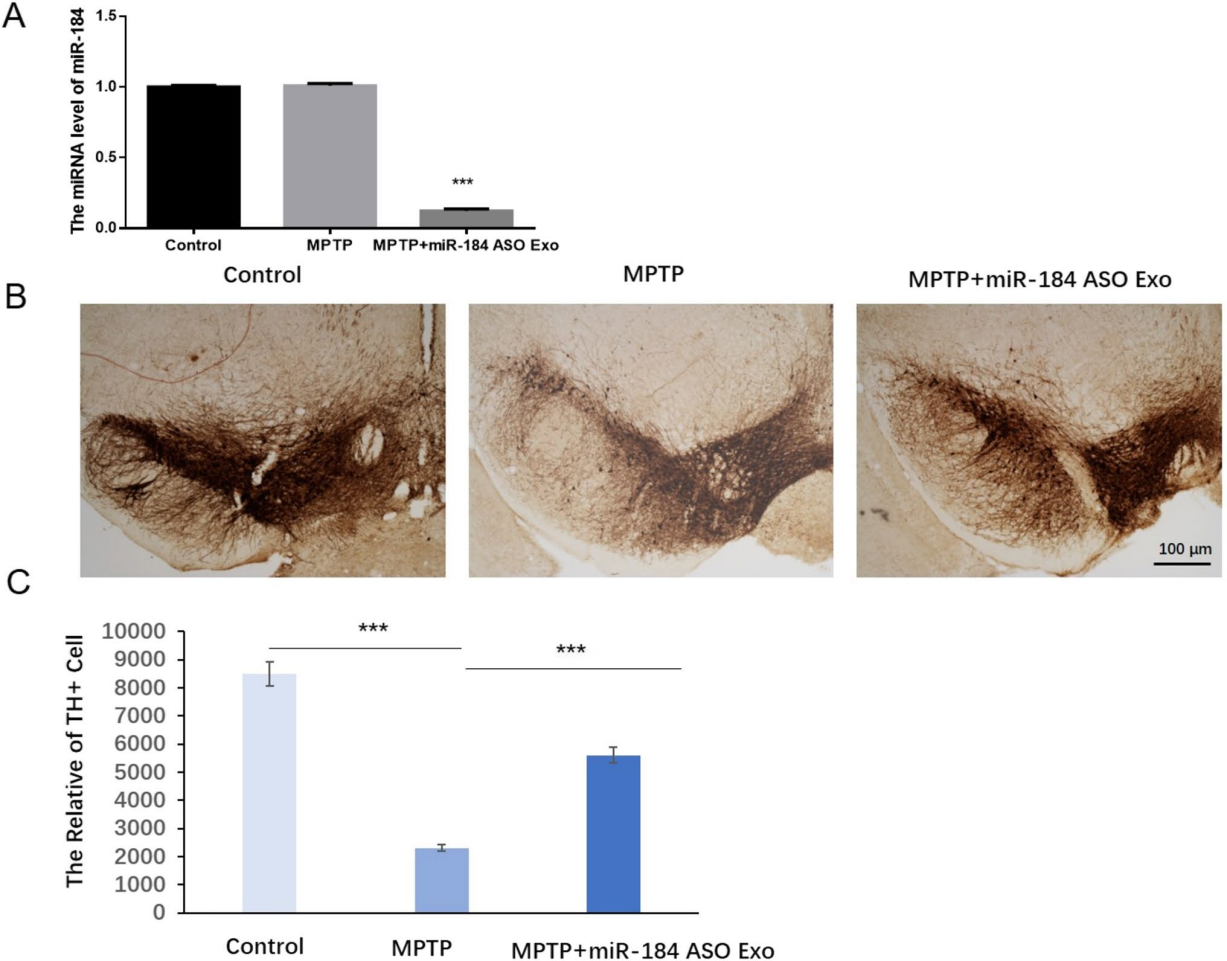
The damage of dopamine neurons in the brain of mice. The miR-184 level after treatment with miR-184 ASO Exo in MPTP-induced mice model. **B** Expression of tyrosine hydroxylase positive neurons in the substantial nigra in control, MPTP, and MPTP + miR-184 ASO Exo groups. **C** Comparison in counts of TH positive cells in mice of all groups. *n* = 3. ***p < 0.001

MiR-184 ASO Exo was injected into the MPTP-induced mice model. As shown in Fig. 7A, the miR-184 level was significantly decreased in MPTP + miR-184 ASO Exo groups compared with control and MPTP groups. TH immunostaining can be used to assess the toxic-damaging effects of MPTP on dopaminergic neurons in mice. The number of TH-positive neurons in brain tissue slices of mice in the model group was significantly reduced compared with that in the control group (Fig. 7B). The TH neurons were counted in the control, MPTP, and miR-184 ASO-injected non-transgenic mice. Induction of miR-184 ASO increased the number of TH-positive neurons in the brain tissue slices of mice (Fig. 7C).

### Injection of miR-184 ASO Exosomes Can Repair Motor Function and Mobility Damaged in the Model Group Mice

**Fig. 8:**
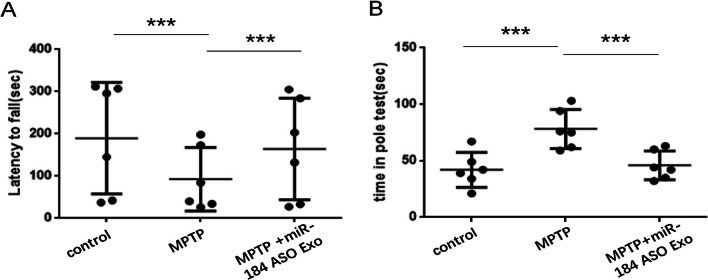
The behavior test in the model group mice. **A** Mice rotarod test results. **B** Mice pole test results. *n* = 6. ***p < 0.001

Conclusions can be drawn from the rotarod test (Fig. 8A) and pole test (Fig. 8B). The motor function of the MPTP group mice was significantly impaired compared with the control group mice. After the injection of miR-184 ASO, we found a marked increase in motor function and locomotor activity.

## Discussion

Neurodegenerative diseases are usually with characteristics of relatively long incubation periods and with neuropathological and neurodegenerative changes before the occurrence of neurological symptoms [42].

MiRNAs were a small RNA molecule that regulated the expression of post-transcriptional genes. The abnormal regulation of miRNA may participate in the progression of the disease by affecting the expression and function of target genes [43]. MiR-184 has been shown to play a role in neurogenesis, chromatin, and histone regulation, which may be a potential target for neurodegenerative diseases [44]. This study showed that the levels of miR-184 in urinary exosomes from PD patients were significantly higher than those of matched healthy control subjects, which were inconsistent with the plasma miR-184 levels in PD patients reported by Na Li et al. [33]. The results of TEM and NTA showed that the number of urinary exosomes increased, and the Brownian movements are slower in PD patients. Overall, compared with urinary exosomes from healthy control, the exosomal distribution in PD patients was relatively condensed and random, and the size was relatively larger by TEM and NTA analysis. The results suggested that urine-derived exosome may be a potential biomarker of PD. CD63 was the surface protein of exosome, and CD63 was equivalent to an internal reference. It may be related to the age of PD patients [45]. This data indicated that we use the same amount of exosome to detect miR-184. Moreover, ZNF865 was detected as the targeted gene of miR-184, which was involved in transcriptional regulation.

Exosomal research is an emerging and rapidly growing field and has increasing attention. Previous research also has shown that blood exosomal levels were increased in depression mice model [46].

There is growing evidence that exosomes may also have a positive impact on neurodegenerative diseases, although some studies showed that exosomes are harmful and may to neurodegenerative diseases [47, 48]. We found that miR-184 ASO could rescue the damaged of motor performance and neuronal apoptosis in PD model.

However, there were some limitation in our study. The number of PD patients was not enough in our study; multicenter research should be carried out to get more numbers and type of PD patients. And the mechanism of miR-184 promoting apoptosis in urine exosomes of PD patients should be considered because there are multiple signaling pathways to promote apoptosis in vivo. Therefore, in the follow-up study, we will further study the biological function and mechanism of miR-184 on apoptosis pathways. Moreover, we will try to screen drugs that regulate the miR-184-ZNF865 signal pathway, which can be injected into the surrounding lesions in vivo to treat PD.

Although studies of EVs are in their infancy stage, previous studies have demonstrated that exosomes have the ability to spread and impede the disease and serve as biomarkers for early diagnosis of PD [49]. Thus, we believe that exosomes may provide a promising role in neurodegenerative disease detection in the future and benefit patients. Based on their inherent features, including feasible access to the brain, exosomes could become a specific drug delivery tool for PD and other neurodegeneration diseases and could also serve as new biomarkers to track the progression of the disease.

## Conclusions

In conclusion, we measured the pathological miR-184 in urinary exosomes of PD patients, which were elevated relative to matched healthy controls. It can be found that the size of urinary exosomes in PD patients is larger than that of healthy controls by TEM and NTA analysis, and the number is more, and the Brownian movements are slower. And miR-184 targeted to ZNF865 and miR-184 ASO could rescue the damage of apoptosis and motor performance in the PD model. These differences are favorable for exosomes to become potential sources of diagnostic markers for PD.

### Supplementary Information

Below is the link to the electronic supplementary material.

Supplementary file1 (JPG 174 KB)

## Data Availability

The datasets generated during and/or analyzed during the current study are not publicly available due to the information protection of patients but are available from the corresponding author upon reasonable request.
