# Supplementary figures and images for: MiR-184 Mediated the Expression of ZNF865 in Exosome to Promote Procession in the PD Model
Source: Mol Neurobiol. 2023 Nov 22;61(6):3397–408. doi: 10.1007/s12035-023-03773-2 (PMC11087344; doi:10.1007/s12035-023-03773-2)

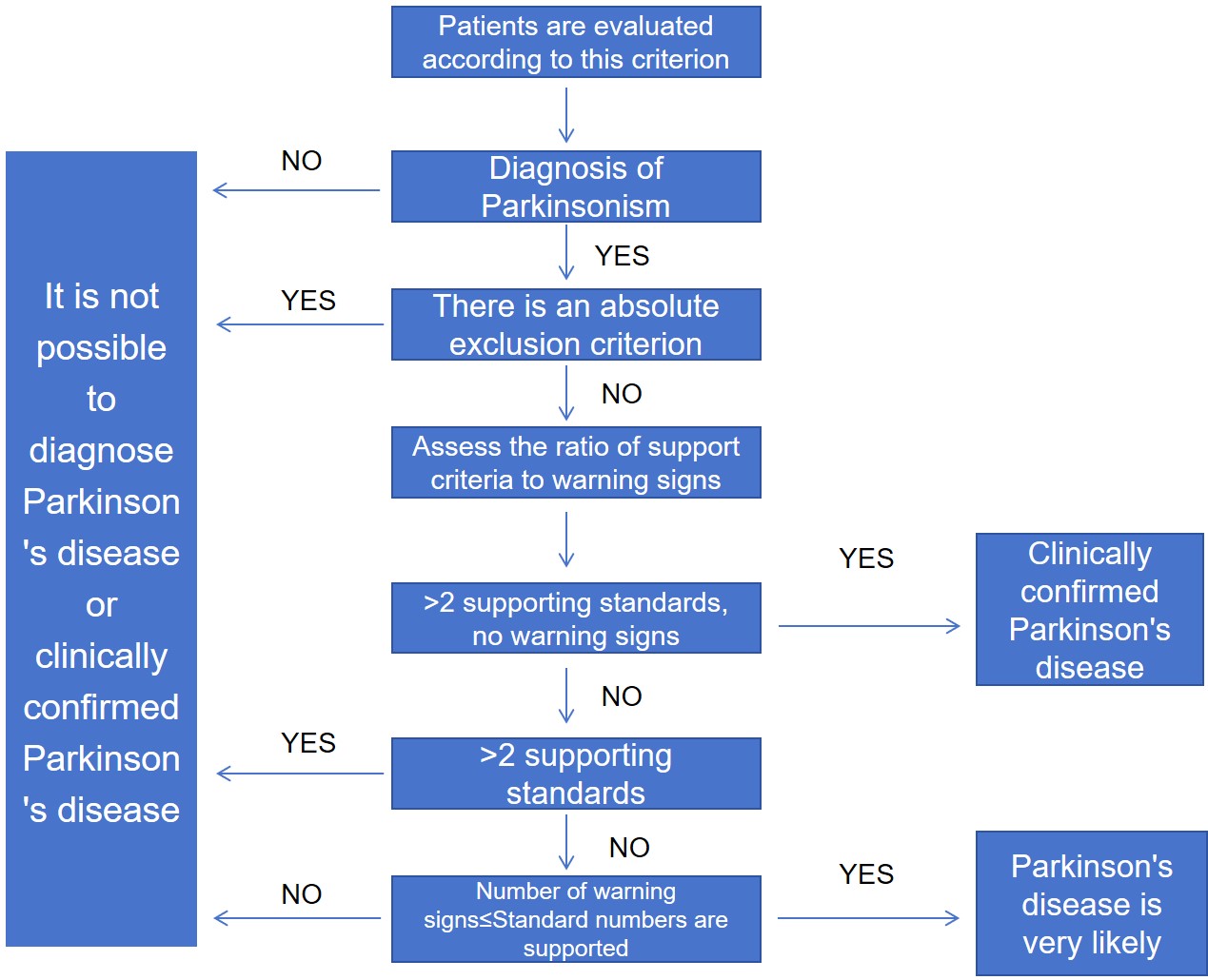

Supplement: Supplementary file 1 — Supplementary file1 (JPG 174 KB) [file 12035_2023_3773_MOESM1_ESM.jpg]
